# Delimiting cryptic species within the brown-banded bamboo shark, *Chiloscyllium punctatum* in the Indo-Australian region with mitochondrial DNA and genome-wide SNP approaches

**DOI:** 10.1186/s12862-021-01852-3

**Published:** 2021-06-16

**Authors:** Fahmi, Ian R. Tibbetts, Michael B. Bennett, Christine L. Dudgeon

**Affiliations:** 1 School of Biological Sciences, The University of Queensland, Brisbane, QLD 4072 Australia; 2 School of Biomedical Sciences, The University of Queensland, Brisbane, QLD 4072 Australia; 3 Research Center for Oceanography, Indonesian Institute of Sciences, Jalan Pasir Putih I No. 1 Ancol, Jakarta, 14430 Indonesia

**Keywords:** Speciation, Species complex, Elasmobranch, Genetics

## Abstract

**Background:**

Delimiting cryptic species in elasmobranchs is a major challenge in modern taxonomy due the lack of available phenotypic features. Employing stand-alone genetics in splitting a cryptic species may prove problematic for further studies and for implementing conservation management. In this study, we examined mitochondrial DNA and genome-wide nuclear single nucleotide polymorphisms (SNPs) in the brown-banded bambooshark, *Chiloscyllium punctatum* to evaluate potential cryptic species and the species-population boundary in the group.

**Results:**

Both mtDNA and SNP analyses showed potential delimitation within *C. punctatum* from the Indo-Australian region and consisted of four operational taxonomic units (OTUs), i.e. those from Indo-Malay region, the west coast of Sumatra, Lesser Sunda region, and the Australian region. Each OTU can be interpreted differently depending on available supporting information, either based on biological, ecological or geographical data. We found that SNP data provided more robust results than mtDNA data in determining the boundary between population and cryptic species.

**Conclusion:**

To split a cryptic species complex and erect new species based purely on the results of genetic analyses is not recommended. The designation of new species needs supportive diagnostic morphological characters that allow for species recognition, as an inability to recognise individuals in the field creates difficulties for future research, management for conservation and fisheries purposes. Moreover, we recommend that future studies use a comprehensive sampling regime that encompasses the full range of a species complex. This approach would increase the likelihood of identification of operational taxonomic units rather than resulting in an incorrect designation of new species.

**Supplementary Information:**

The online version contains supplementary material available at 10.1186/s12862-021-01852-3.

## Background

The development of genetic and genomic studies has substantially influenced how species are defined taxonomically, with a shift from traditional taxonomy based on morphological and biological characters to one that includes or entirely relies on DNA-evidence (e.g. [1]). While the necessity for phylogenetic support in defining a species is the subject of debate, many journals require phylogenetic analyses in the description of new taxa [2]. Advanced DNA sequencing has revealed species complexes and cryptic species previously assumed to be subspecies or populations of a single species through traditional taxonomic analysis [3–5]. In evolutionary theory, species complexes are considered recently diverged from evolving metapopulations [3, 6]. As an ongoing process of speciation, morphological differentiation may develop later due to local adaptation to new environments [3, 4]. Cryptic species may be present within a complex when morphologically indistinguishable individuals are revealed to be genetically distinct [6–8]. While there are disparities in the definition of cryptic species in terms of their biology, they provide challenges for taxonomic and evolutionary studies [9]. For instance, the discovery of cryptic species in what was previously considered a single wide-ranging species raises questions whether geographically-structured populations can be differentiated from different species within the same region, leading to taxonomic confusion [10, 11]. A reliance on genetic evidence alone to split a species complex into two or more new species presents wildlife managers and ecologists with challenges as morphological features that facilitate visual discrimination are absent [12–14]. A lack of field-based identification may confound determination of a species’ conservation status, and management strategies that might be relevant for that species [6, 15, 16].

Cryptic species are common in many groups of taxa and various habitats, including in marine environments [4, 6, 17]. In elasmobranchs, the number of recognised cryptic species has increased substantially within the last decade, primarily due to a large, multi-taxa, phylogenetic study of mitochondrial NADH2 sequences [18]. Several putative cryptic species have been identified with most originating from areas of high geographic complexity, and in taxa with low dispersal ability [18, 19]. The use of genome-wide approaches alongside mitochondrial markers has improved the ability to identify cryptic diversity of elasmobranchs (e.g. in mobulids [20]).

**Fig. 1:**
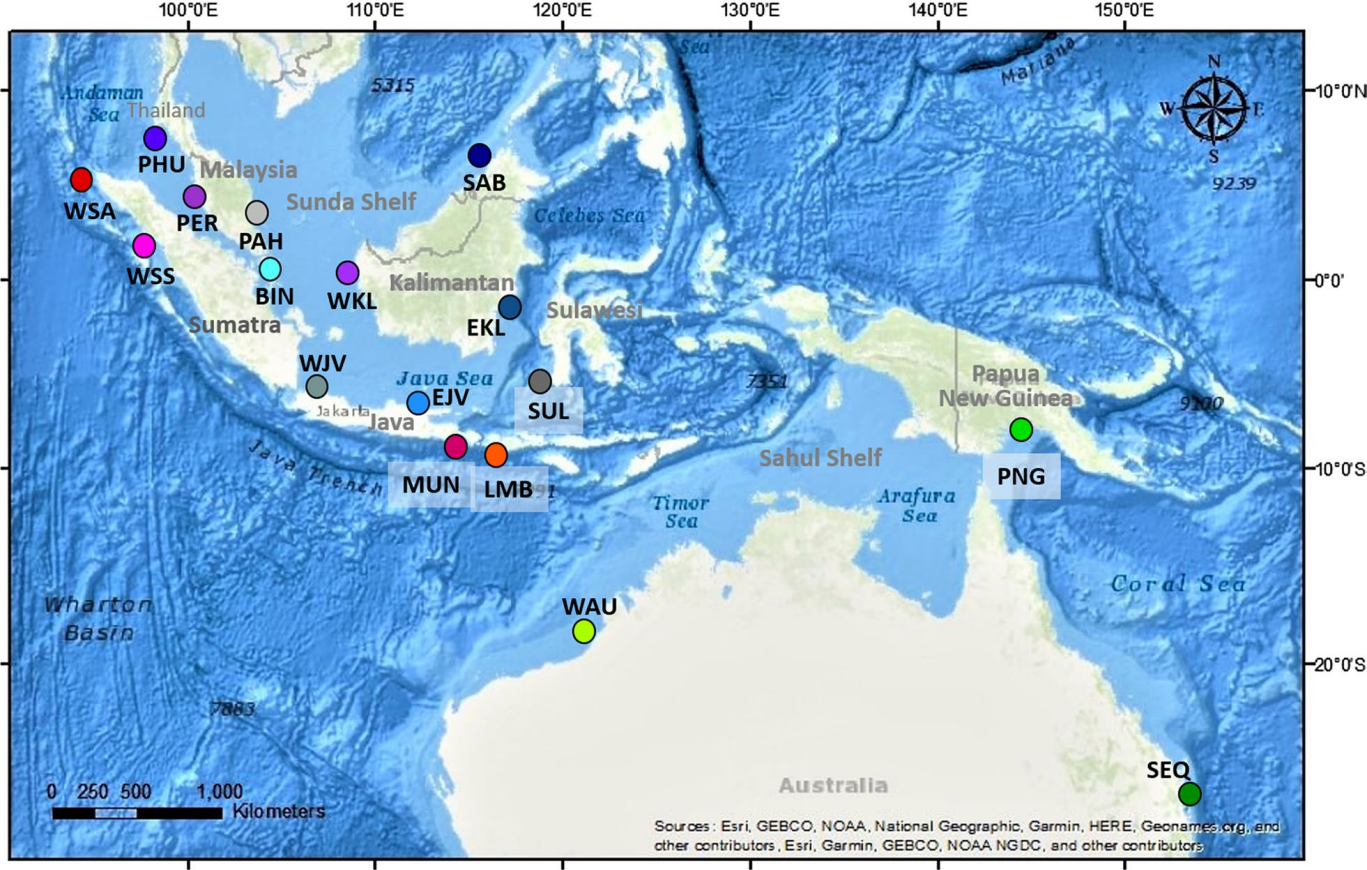
Map showing the *Chiloscyllium punctatum* sample-collection locations. Abbreviations of location names refer to Table 2

In this study, we examined the brown-banded bambooshark, *Chiloscyllium punctatum*, a species distributed in near-shore environments within the Indian and the Western Pacific Oceans [21–25] (Fig. 1), and the most commonly caught shark in coastal fisheries in Southeast Asia [26]. Previous genetic and morphological studies have suggested that *C. punctatum* contains two cryptic species [18, 27]. However, the genetic study only included a few sampling locations with uneven sample sizes and large geographic breaks, likely under-representing the species’ genetic diversity within the region [17]. Here we use genome-wide nuclear single nucleotide polymorphisms (SNPs) along with mitochondrial DNA lineages to assess potential cryptic speciation within an extensive, previously un-sampled area within the Indonesian archipelago, and to evaluate the use of genetic and genomic data to delineate species.

## Results

### Mitochondrial gene phylogeny

**Fig. 2:**
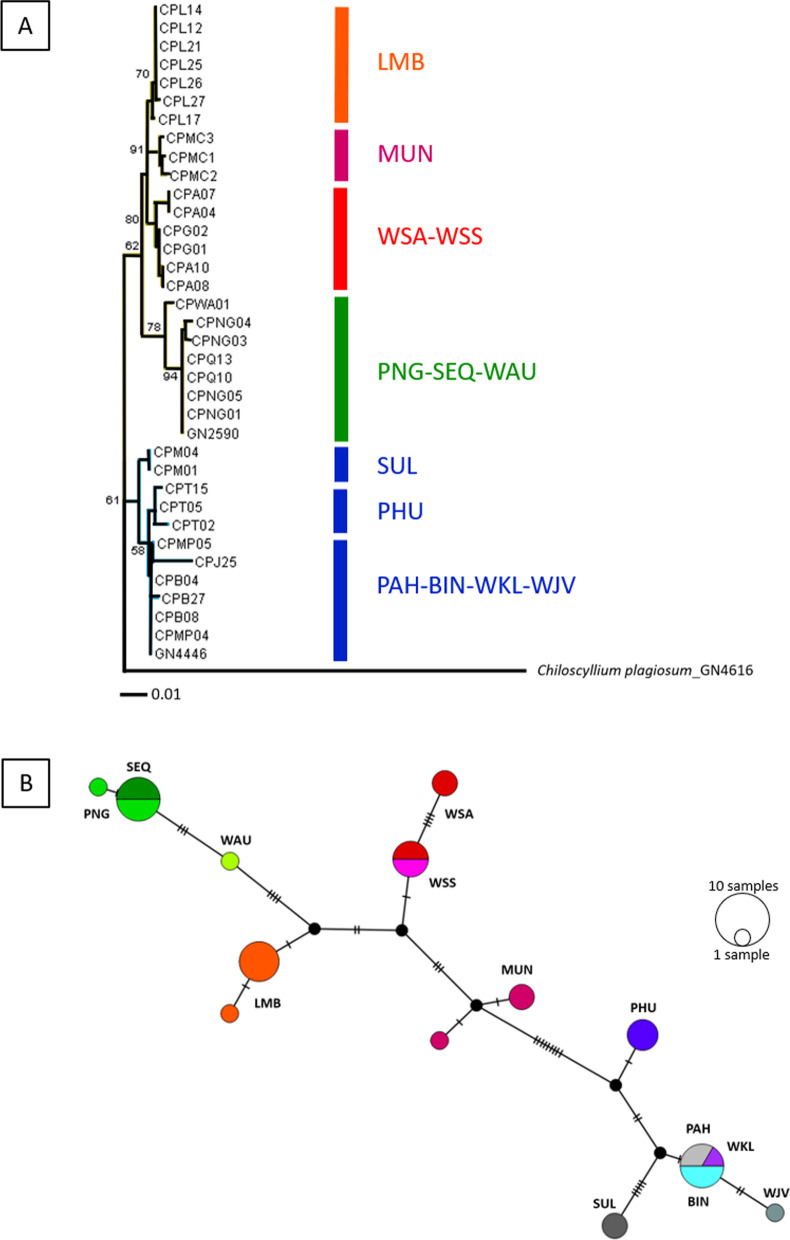
**A** Maximum likelihood (ML) tree based on the Tamura-Nei model analysis (TN93 + G) of the NADH2 data with bootstrap support for 1000 replicates. The outgroup is the congener species, *Chiloscyllium plagiosum* from West Kalimantan; **B** Median-joining haplotype network of *Chiloscyllium punctatum* from Indo Australian region. Circle diameter represents haplotype frequency, connecting lines represent single mutational steps, and hash marks represent the number of haplotypes

In total, 34 individuals were sequenced for the mitochondrial NADH2 marker across 12 locations. Two phylogenetic analyses of the trimmed alignment sequence data (1044 bp) (maximum likelihood phylogenetic tree and median-joining haplotype network) showed similar clusterings of haplotypes (Fig. 2). The phylogenetic tree indicated two major clades: (i) samples from Papua New Guinea (PNG), Australia (SEQ and WAU), Lombok (LMB), west coast of Sumatra (WSA and WSS), Muncar (MUN); and (ii) samples from South East Asia including Thailand (PHU), Malaysia (PAH) and Indonesia (BIN, WKL, WJV, SUL). Within the first clade, samples from Papua New Guinea and Australia formed a distinct cluster (Australian region) separated from samples from the south coast of Indonesia (the Indian Ocean region). Within the second clade, samples from the Indo-Malay region (PAH, BIN, WKL and WJV) formed a cluster separated from Thailand (PHU) and samples South Sulawesi (SUL) (Fig. 2A). The mean pairwise genetic distances (*d*) between Indo-Malay samples and the Australian region ranged from 0.0264 to 0.0462, while the pairwise distances within the Indo-Malay region ranged from 0.0008 to 0.0229 (see Additional file 1). Specimens from the west coast of Sumatra were more genetically similar to Lombok and Muncar (southern Indonesia), although located closer geographically to the Indo-Malay region (see Fig. 1).

The median-joining haplotype network showed fine-scale groupings of the 13 haplotypes, with two main haplogroups between samples from the Indo-Malay region and those from the Indian Ocean and Australian regions. Within the Indo-Malay grouping, samples from PAH, BIN and WKL formed a large haplotype, separated from WJV by 0.2% of sequence divergence. While LMB, MUN, PHU and SUL formed a sub-cluster of haplotypes. Samples from the west coast of Sumatra (WSA and WSS) formed a distinct haplotype, while Papua New Guinea (PNG) and Australia (SEQ and WAU) formed an Australian region cluster (Fig. 2B). The largest haplotype divergence (1.7–2.8%) occurred between the Indo-Malay samples and those from the Australian region. Samples from the Indian Ocean region (WSA, WSS, LMB and MUN) formed distinctive haplotypes separated from the Australian region by 0.5–1.4%. In contrast, samples from South Sulawesi (SUL) formed a different sub-cluster of the Indo-Malay haplotypes by 0.6–0.8%.

### SNP genotyping

**Fig. 3:**
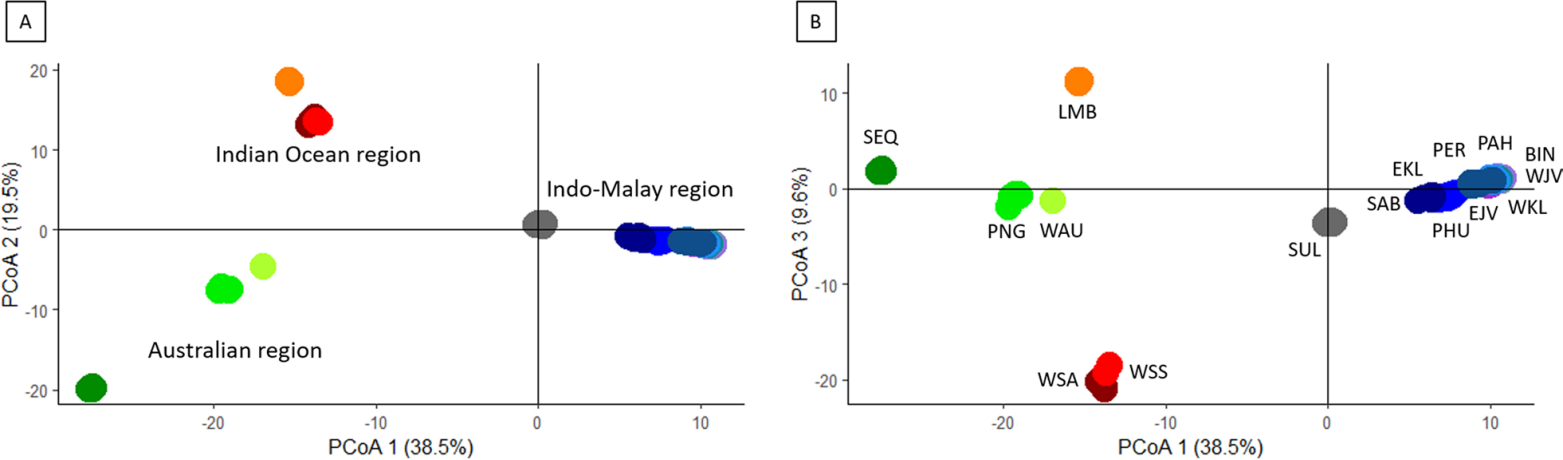
Principal Coordinate Analysis plots of *Chiloscyllium punctatum* from the Indo-Australian region of 6,099 SNPs based on: **A** axis 1 *vs* axis 2, and **B** axis 1 *vs* axis 3

A total of 82,994 SNPs were detected from 148 individuals across 16 sampling locations. Filtering criteria resulted in the subsequent reduction of SNP loci to 6,099 SNP loci (see Additional file 2) that was used for the population structure and phylogenetic analyses. All individuals clearly grouped into their geographical regions. A PCoA based on axis 1 (38.5% of the variation among individuals) and axis 2 (19.5%) showed three main clusters for the *C. punctatum*-complex (Fig. 3A): the Indo-Malay region, the Australian region and the Indian Ocean region (WSA, WSS, LMB). However, axis 3 (9.6%) showed a clear separation of LMB from the WSA-WSS group (Fig. 3B). Individuals from South Sulawesi (SUL) showed some separation from the Indo-Malay region. Separation of individuals from SEQ, and those from WAU and PNG was evident within the Australian region (Fig. 3A, B).

**Fig. 4:**
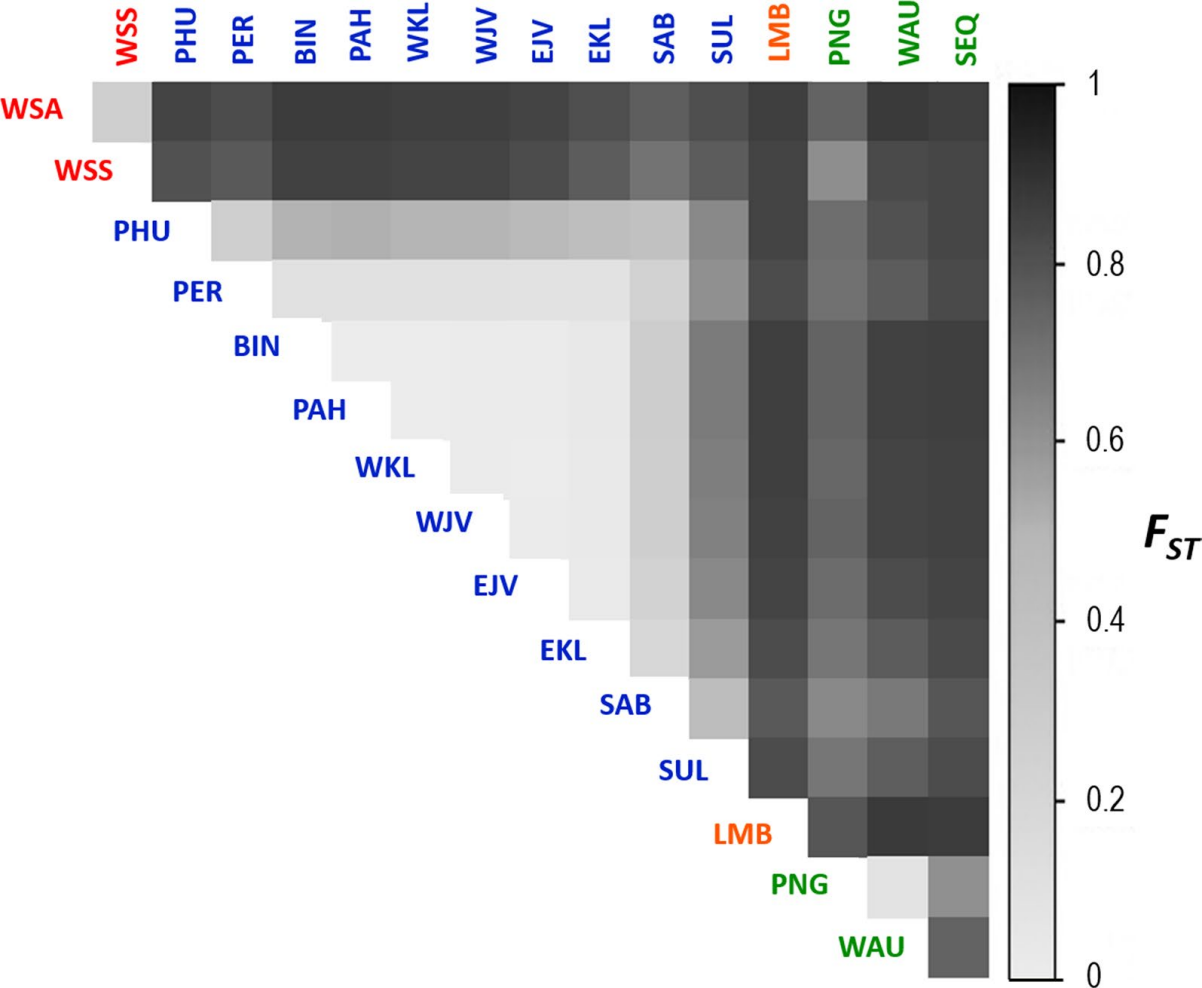
Heatmap showing the pairwise *F*_*ST*_ values among locations based on SNP data

Pairwise *F*_*ST*_ analyses resulted in significant structuring among locations but the magnitude of differentiation varied by several orders (*F*_*ST*_ values = 0.001 –0.876, *P* = 0 – 0.1). Some locations within the Indo-Malay region (BIN, PAH and WKL) showed limited structuring among locations (*F*_*ST*_ ≤ 0.1, *P* > 0.05). In contrast, other locations within the region (PHU, PER, WJV, EJV, EKL and SAB) showed strong population structure (*F*_*ST*_ ≤ 0.5, *P* < 0.05). Conversely, samples from the west coast of Sumatra (WSA, WSS) and Lombok (LMB) showed distinct genetic divergence (*F*_*ST*_ > 0.5, *P* < 0.05) from those within the Indo-Malay region and the Australian region (Fig. 4, Additional file 3).

### Phylogenetic inferences

There were no fixed differences among locations in the Indo-Malay region except for South Sulawesi (SUL), which showed minimal fixed differences of 1–3%. The west coast of Sumatra (WSA) had fixed allele differences of 10–22% with all other sampling locations. A similar pattern was observed in individuals from Lombok (LMB), which differed from all other locations by 10–21%. The Australian region differed from all other locations by 6–23% fixed differences but showed low fixed differences within the region (1–2% fixed differences) (see Additional file 4).

**Fig. 5:**
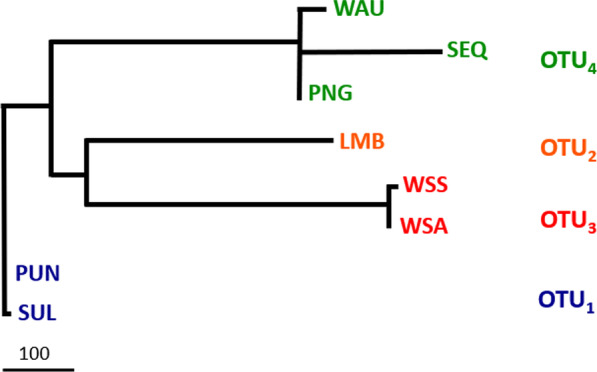
Unrooted phylogenetic tree of four putative OTUs of *C. punctatum* based on Fitch-Margoliash maximum likelihood inferred from fixed allele difference analysis. Scale bar represents the number of allele differences

Two amalgamation steps were undertaken based on fixed allele differences. Firstly, only locations with no fixed differences resulted in all individuals from the Indo-Malay region (BIN, PAH, PER, WKL, WJV, EJV, EKL, SAB, and PHU) being pooled into one group (called PUN). Few but no significant differences in each comparison (*p* > 0.05) combined South Sulawesi (SUL) with the Indo-Malay group (PUN) but Lombok (LMB) and the west coast of Sumatra (WSA, WSS) remained separated. Individuals from PNG and WAU grouped together with SEQ into the Australian region group. The final result was four group entities with absolute fixed-differences, and we designated them as four distinct operational taxonomic units (OTUs) (see Additional file 5). The distance tree generated from the Fitch-Margoliash distance analysis combined the west coast of Sumatra (OTU_2_) and Lombok (OTU_3_) into a single clade (Fig. 5).

**Fig. 6:**
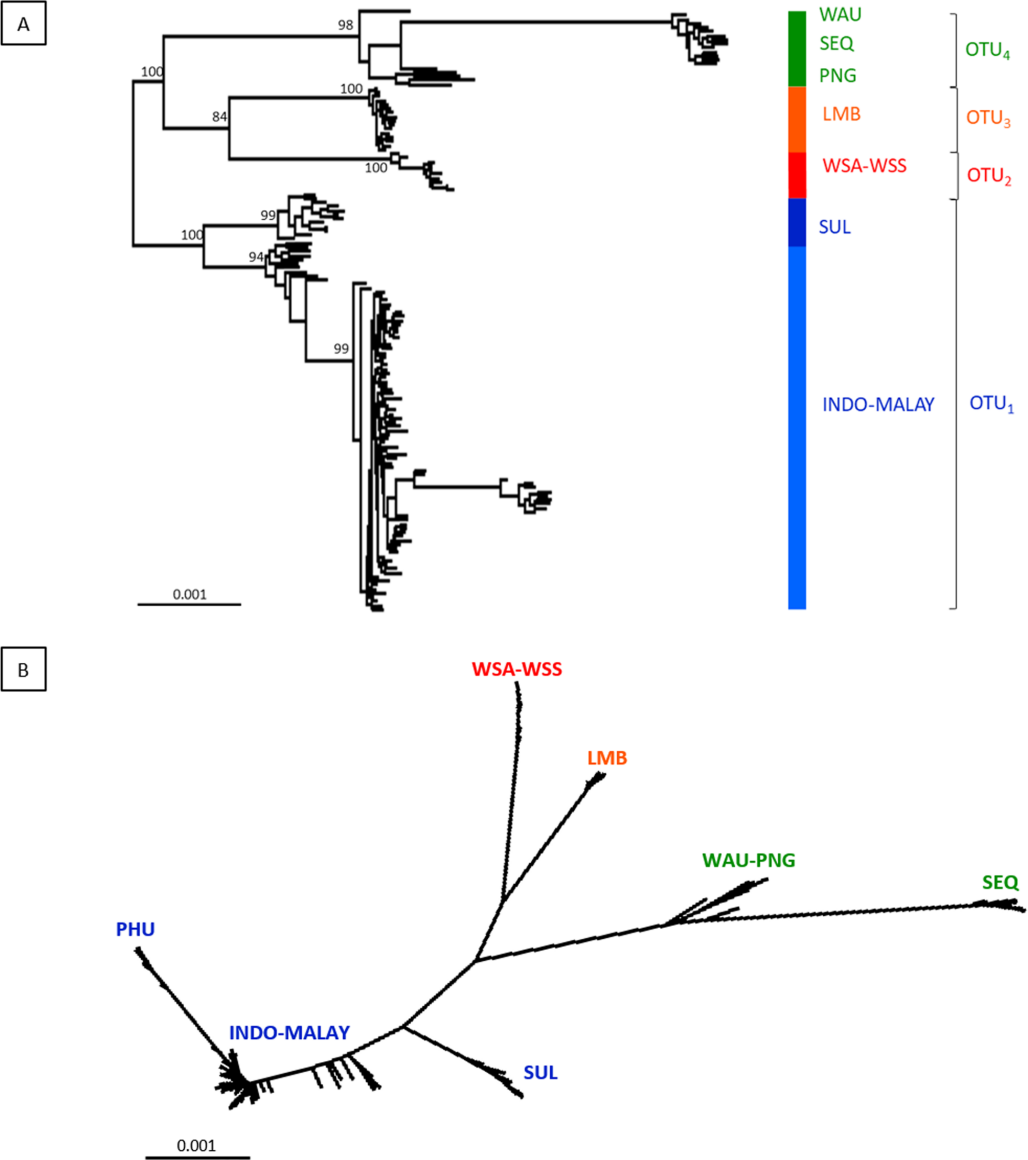
Phylogenetic tree of *Chiloscyllium punctatum* inferred from the concatenated sequence fragments of 6099 SNPs data using RAXML based on **A** maximum-likelihood tree; **B** a radial phylogram. Numbers on branches represent bootstrap values from 1000 replicates. Only bootstrap values of 80% or higher are presented

The maximum-likelihood tree inferred with the RAXML for 148 individuals with 381,018 bp of concatenated SNP sequence revealed three distinct clades. Most individuals from the Indo-Malay region, with the exception of SUL, formed a clade, while LMB, WSA and WSS formed a separate clade (Fig. 6A). Although individuals from the Australian region occur in the same clade, individuals from SEQ were clearly separated from WAU and PNG in a longer branch (Fig. 6B).

### Species delimitation

**Fig. 7:**
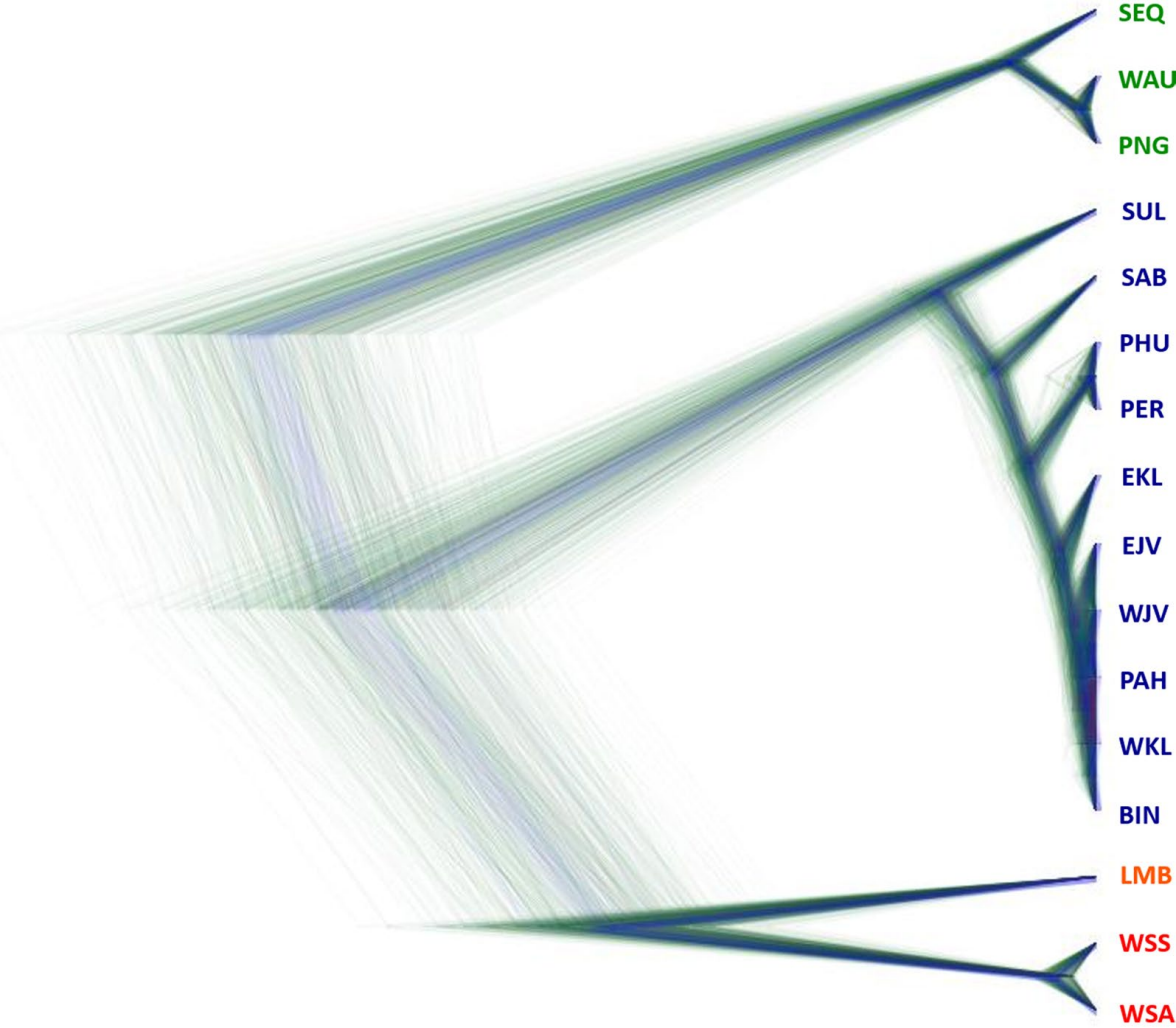
The species trees of 20 individuals representing 16 sampling locations from SNAPP analysis taken from every 1000 steps of the total 2,000,000 iterations, visualised by DensiTree software

The Bayesian coalescent-based species tree derived from the SNAPP analysis divided *C. punctatum* into four distinct clades corresponding to the OTUs. Relationships among locations within clades remained consistent in all topologies (Fig. 7 and Additional file 6). The Bayes factor delimitation (BFD*) analysis supported the hypothesis to delimit *C. punctatum* into four putative species based on OTUs as the top-ranked model (H-5) with the estimate of the highest value of the marginal likelihood with a decisive Bayes factor (2log_e_ BF > 10) against the initial model (H-1) [28] (Table 1).

**Table 1:** The Bayes factor delimitation (BFD*) analysis for the five species delimitation models of *Chiloscyllium punctatum* with the rank based on the marginal likelihood estimates (MLE)

Model	Species	MLE	Rank	BF
H-1: (Indo Malay + Indian Ocean) *vs* (Australian region)	2	− 44,816.57	4	–
H-2: (Indo Malay) *vs* (Indian Ocean + Australian regions)	2	− 43,468.79	3	− 2695.58
H-3: (Indo Malay + Sumatra) *vs* (Lombok + Australian region)	2	− 45,849.86	5	− 2066.56
H-4: (Indo Malay) *vs* (Indian Ocean) *vs* (Australian region)	3	− 37,765.24	2	14,102.68
H-5: (Indo Malay) *vs* (Sumatra) *vs* (Lombok) *vs* (Australian region)	4	− 35,340.07	1	18,953.01

Two models show positive Bayes factor (BF) against the initial model H-1

### Biological characteristics

**Fig. 8:**
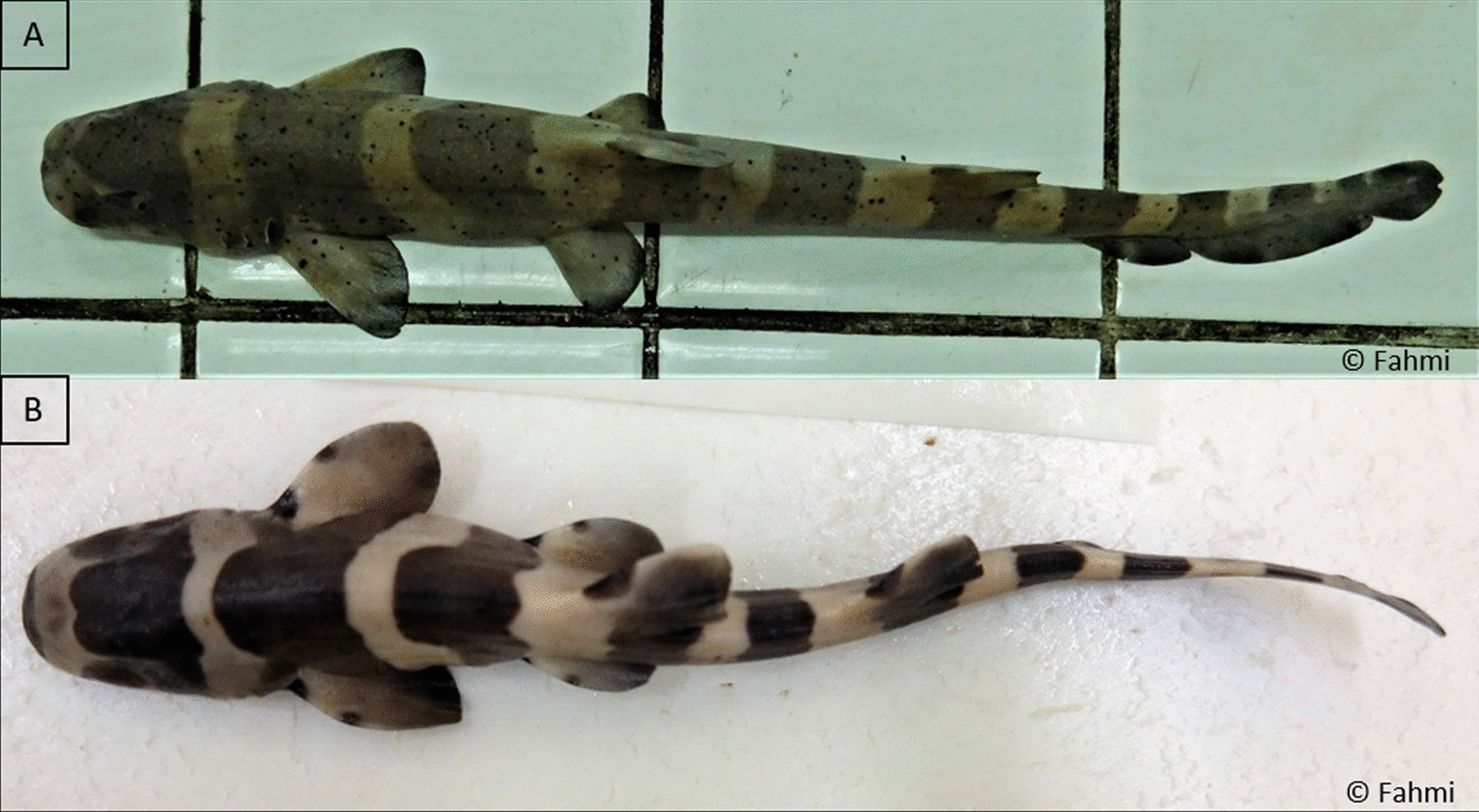
Juveniles of *Chiloscyllium punctatum* from: **A** West Java, Indonesia (OTU_1_) and **B** Papua New Guinea (OTU_4_)

Maximum *TL* measurements from each location supported the Indo-Malay species (OTU_1_) as different from the other OTUs (see Table 2) with all individuals from OTU_1_ reaching their maximum *TL* at a smaller size (< 110 cm *TL*) compared with those from OTU_2,_ OTU_3_ and OTU_4_ (> 110 cm *TL*). Based on available references, *C. punctatum* attains maturity at ≤ 100 cm *TL*, with individuals from Indo-Malay region (corresponding to OTU_1_) mature at around 67–70 cm *TL* [29, 30], while those from Australia and PNG (corresponding to OTU_4_) mature at about 84–87 cm *TL* [24, 31]. No information on size at maturity is available for the regions that fall within OTU_2_ and OTU_3_. In addition to differences in body size, there are some morphological differences between individuals from OTU_1_ and other OTUs. Juveniles of OTU_1_ have 11 dark bands and numerous dark speckles on the body, while those from other OTUs have 10 dark bands (OTU_3_ and OTU_4_) or 12 dark bands (OTU_2_), and lack of dark speckles (Fig. 8). Nevertheless, those biological features provide an indication of differences among OTUs that support the genetic findings.

**Table 2:** Data relating to *Chiloscyllium punctatum* samples from the Indo-Australian region

Location	Code	Country	OTU	Fishing ground	*n*	*TL* (cm)	Max *TL* (cm)
Phuket	PHU	Thailand	OTU_1_	Andaman Sea, upper Malacca Strait	9 (3)	47.2–84.5	91^1^
Perak	PER	Malaysia	OTU_1_	West coast of Malaysian Peninsular	11 (–)	43–90	100^2^
Pahang	PAH	Malaysia	OTU_1_	East coast of Malaysian Peninsular	10 (2)	47–83	103^2^
Sabah	SAB	Malaysia	OTU_1_	North of Borneo	10 (–)	49.3–98	105.6^2^
Bintan	BIN	Indonesia	OTU_1_	Malacca Strait	10 (3)	65–80	80
West Kalimantan	WKL	Indonesia	OTU_1_	West coast of Kalimantan	10 (–)	70–95	99^3^
Aceh	WSA	Indonesia	OTU_2_	Northwest coast of Sumatra	8 (4)	78–98	146^5^
Sibolga	WSS	Indonesia	OTU_2_	West coast of Sumatra	2 (2)	64.9–71.6	113^4^
West Java	WJV	Indonesia	OTU_1_	Seribu Islands waters	11 (1)	36–83	93^3^
East Java	EJV	Indonesia	OTU_1_	Java Sea	10 (–)	44–90	90
East Kalimantan	EKL	Indonesia	OTU_1_	East coast of Kalimantan	10 (–)	52–92	92
South Sulawesi	SUL	Indonesia	OTU_1_	Southwest of Sulawesi	12 (2)	50–90	99^3^
Muncar	MUN	Indonesia	–	Bali Strait	(3)	57–90	105.5^3^
Lombok	LMB	Indonesia	OTU_3_	Lombok Strait	16 (7)	80–132	148^3^
South PNG	PNG	Papua New Guinea	OTU_4_	Gulf of Papua	5 (4)	32–81	132^6,7^
Western Australia	WAU	Australia	OTU_4_	West coast of Australia	1 (1)	59	132^6,7^
South East Queensland	SEQ	Australia	OTU_4_	Moreton Bay	13 (3)	62.2–102.3	132^6,7^

Data collected from: ^1^DoF Thailand; ^2^SEAFDEC; ^3^LIPI; ^4^Dharmadi et al. [78]; ^5^WCS Indonesia; ^6^White et al. [31]; ^7^Last and Stevens [24]. All other samples collected as part of this study (2018–2019). n = number of tissue samples analysed for SNPs and mtDNA (in parentheses); TL = total length of individuals used in the study; Max TL = recorded maximum TL from each location

## Discussion

### Species-population boundary

Both mitochondrial DNA and genome-wide SNP data analysis support a species complex in the brown-banded bamboo shark. By sampling comprehensively across a part of its range, we have revealed greater genetic variation and potentially more species than suggested by a previous study [18] that included fewer sampling locations. Obtaining representative samples across a species’ distributional area can be challenging for elasmobranchs. There are potential difficulties in obtaining a comprehensive sample set for a species, due to rarity in nature or in markets; difficult to catch; financial cost; large specimen sizes; wide geographical spread and species protections, which limits access to samples or requires special permits [32]. Still, many new species of elasmobranch have been described based on limited geographic sampling over the last decade (e.g. [33–35]).

Our analyses on the genetic data of *Chiloscyllium punctatum* support the hypothesis of Naylor et al. [18] that a potential cryptic species occurs in Australia. However, by including a more comprehensive suite of sampling sites that captured more genetic variation, we were able to identify four OTUs within *C. punctatum*. Yet, as the OTUs are allopatric, it is difficult to discern the population-species boundary on genetic data alone. Depending on the nature of the supporting information, *C. punctatum* species complex may consist of three or four putative species. Based on the genetic data alone, the four OTUs can be interpreted as four distinct species supported by both phylogenetic and species delineation (BFD*) analyses.

The type locality for the original description of *C. punctatum* was described from a geographic location associated with OTU_1_ in the waters off Jakarta, Indonesia [36]. Specimens from Jakarta were clearly grouped in OTU_1_, which comprises individuals that inhabit shallow waters (20–50 m) within the Indo-Malay Archipelago [22, 23]. Individuals from OTU_1_ are not only genetically separated to those in the other OTUs, but the distinction is also supported by biological, ecological and geographical data. Individuals from OTU_1_ have a relatively smaller body size with the maximum *TL* at around 1 m*,* compared with other OTUs that have a maximum *TL* more than 130 cm (see Table 2). That Indo-Malay individuals mature at a smaller size than do those from Australia further supports species delimitation [24, 27, 30, 31], as well as other detailed information on morphometric and meristic characters for specimens from those regions (Fahmi et al., unpublished data). However, there is still a lack of information on size at maturity and detailed morphological characters for individuals from the Indian Ocean (OTU_2_ and OTU_3_). Therefore, further studies are needed to explore biological features for species delimitation from those regions. Ecologically, we observed that *C. punctatum* from the Indo-Malay region is commonly found on soft-bottom habitats (e.g. sand flat, muddy substrate). Geographically, all locations that were linked by shallow waters within the Sunda Shelf region (from Phuket, Thailand to East Kalimantan, Indonesia) showed strong connectivity except for South Sulawesi, which is geographically the furthest east location included in OTU_1_. South Sulawesi is also separated from the Indo-Malay region by a deep trench that could restrict gene flow.

Individuals from the Indian Ocean and Australian regions may be grouped into a putative species based on similarities of their biological characters, such as the larger maximum body size than for the Indo-Malay region, or may be separated into two distinct species based on ecological and geographical perspectives. However, the BFD* analysis lent support to two separate groups. Therefore, individuals from Australian (OTU_4_) and the Indian Ocean regions (OTU_2_, OTU_3_) can be designated as an incipient species if there are any supportive morphological characters that can distinguish them.

In the Indian Ocean region, individuals from the west coast of Sumatra (OTU_2_) were clearly separated from Lombok (OTU_3_) based on their fixed allele differences. However, this may reflect population level difference, given continuity of habitat between their geographic locations in the outer margin of the Sunda Islands region. Further, the mtDNA phylogram grouped both locations in the same clade together with those from Muncar in the south of the Bali Strait (see Fig. 2A). The high fixed allele differences, *F*_*ST*_ values and marked separation in the phylogenetic tree of specimens between Lombok and other locations may be caused by the unique geographical position of this location in the Lesser Sunda Island region. Lombok is separated from other locations by deep water (The Bali Sea and the Flores Sea in the north, and the Indian Ocean in the south, each with depths of ca. 1500–3000 m) and the strong Indonesian Throughflow current in the west (the Lombok Strait). Together, these likely represent significant barriers to gene flow between Lombok and both the Indo-Malay and the Australian regions. The geographical barrier in the Lombok Strait is an important feature for speciation in particular groups such as the separation of two blue-spotted maskray species, *Neotrygon caeruleopunctata* in the west and *N. australie* in the east [37, 38]. In contrast, this barrier is just resulting in population structure for other species such as the Indonesian wobbegong, *Orectolobus leptolineatus* [33] and Indonesian speckled catshark, *Halaelurus maculosus* [39] that occur along the eastern Indian Ocean. Additional sampling at locations along the south coast of Java and the west coast of Sumatra is required to bridge the geographic gap that exists in our data between Sumatra and Lombok to further resolve whether the separation demonstrated here is representative of speciation or population differences. Nevertheless, in terms of habitat preference, *C. punctatum* from along the west coast of Sumatra, south of Java to Nusa Tenggara in eastern Indonesia (including Lombok), inhabit similar coral and rocky reef habitats.

The bamboo shark species from the Australian region (OTU_4_) can be found in various habitats, including coral reefs, seagrass beds, mangroves and estuaries [24]. The tectonic plate boundary between the Sahul Shelf (South PNG, Western Australia, and South East Queensland) and the Sunda Shelf (Greater and Lesser Sunda Islands) is considered a major barrier causing a genetic break that separates populations and potential species within this complex. Examples of the influence of this barrier on species level differences can be found in blackspot sharks (*Carcharhinus dussumieri*) and banded eagle rays (*Aetobatus narinari*) [40, 41]. Even though species in the Australian region formed a distinct clade, there are some fixed differences between specimens from South East Queensland and those from Western Australia and South PNG. Nevertheless, there is no physical barrier, such as deep water or strong currents that might be a driver of genetic segregation between these locations. Therefore, we suggest that locations in OTU_4_ represent a single species with population structure, and the differences within the clade may reflect a variation by distance effect [42]. This should be further tested by intermittent sampling locations in northern Australian waters. The difference in the phylogenetic results between mtDNA and SNP data for individuals within the Australian region, where individuals from SEQ showed a separation from PNG and WAU based on SNP analysis but showing no differences in mtDNA, may be an artefact of the level of sensitivity of each marker used. Discrepancies between genetic markers are commonly found in elasmobranchs [18, 43, 44], such as in *Carcharhinus limbatus* [45] and *Hemiscyllium ocellatum* [18, 46]. Nevertheless, further studies are also needed to identify the possibility of reproductive isolation among locations due to the limited dispersal behaviour of the bamboo shark.

Our study also revealed that geographic distance does not directly correspond to genetic distance. For instance, Aceh (OTU_2_) is separated from Phuket (OTU_1_) by only about 470 km while some sites within the OTU_1_ cluster (such as between Phuket and West Java) are separated by more than three times the distance. Similarly, Muncar and East Java are separated by only 300 km and connected by the narrow and shallow waters of the Bali Strait, yet showed strong genetic separation. A possible reason for that separation is habitat type. In addition to the role that deep water and tectonic plate barriers play, habitat preferences are likely to be important in either structuring populations or delimiting species [47, 48]. Based on habitat preferences, the four OTUs can be further delimited into three groups with OTU_2_ and OTU_3_ combined. The BFD* analysis also lent high support to this hypothesis.

The decision to delimit species complexes may vary among taxa. In some taxa, genetic differences may not be reciprocal with either morphology (phenotypic plasticity), biology (reproductive traits), or ecological characteristics (habitat preferences) [49]. In elasmobranchs, genetics has been used to delimit cryptic species in a species complexes in the genera *Carcharhinus*, *Aetobatus* and *Neotrygon* [38, 40, 50, 51], as well as coalesce two genera into one genus due to genetic indistinctiveness, such as in *Mobula* [52].

For some taxonomic groups such as birds and some mammals, the term 'subspecies' is used to define a population or group of populations that are distinctive yet insufficiently different to constitute a separate species based on subtleties in appearance and/or in genetic makeup [53–55]. The term 'subspecies' is also used to differentiate a species complex based on ecological speciation for populations without multigene discontinuity [56]. Moreover, 'subspecies' is applied to geographically isolated populations driven by biodiversity and conservation purposes, such as in some freshwater fishes [54]. However, the use of subspecies in some taxa is not preferable due to confusion with the population term [57, 58], and has been rarely used in the past few decades for marine fishes [59]. For elasmobranchs, this term has only been applied to few taxa, e.g. in catsharks [60–63], dogfishes [64], smooth-hounded sharks [65], hammerheads [66], eagle rays [67], and skates [68]. Some of those subspecies remain valid such as the smooth-hounded shark (*Mustelus canis canis* and *M. c. insularis*) and for several species of skates (from Genus *Raja* and *Leucoraja*), while others were considered junior synonyms or have been elevated into distinct species [69]. Therefore, the use of subspecies for the bamboo shark OTUs is plausible if they cannot be definitively classified at either the species or population level.

### Conservation implications of the species delimitation

Splitting cryptic species in a complex into one or more distinct species may provide advantages for the species, not just for formal scientific recognition, but also to assess conservation risk [3, 4, 6, 70]. Thus, from a conservation perspective, separating *C. punctatum* into two or three species is desirable as differences in biological features, spatial distribution, habitat occupancy, and type of fishery that operates in an area could influence how each species should be managed for sustainability. For instance, due to the intensive trawl operations in the Indo-Malay region [26, 71], the species based on OTU_1_ would be subjected to higher fishing pressure than individuals within OTU_2_ and OTU_3_ where bottom longline fisheries operate due to unsuitable substrates for bottom trawling_,_ and compared to OTU_4_ where they are neither targeted nor caught as bycatch. This situation may lead to differences in threat profiles that necessitate revision among the OTUs of their conservation status.

In terms of fisheries management, each OTU with distinct fisheries characteristics can be treated separately. For instance, limits on minimum size or permitted fishing gear may differ between OTUs due to the nature of the fishery. For countries that implement ecosystem or species-based conservation management such as in Indonesia (referring to Regulation of the Minister of Forestry P.57/Menhut-II/2008 and Regulation of the Minister of Marine Affairs and Fisheries 3/2010), regulating two different options of fisheries management for one species is challenging, compared with countries that implement conservation strategies on a population-basis. Therefore, splitting *C. punctatum* into at least two or three different species for management purposes is appropriate for countries that apply species-based management, as long as diagnostic morphological characters are available. Especially when they are marketed in the same place with a lack of traceability, as occurs in Indonesia.

In contrast, splitting a cryptic species complex into several species based purely on genetics can cause problems if there are no strong supportive diagnostic characters to differentiate them in the field. An example is in the *Neotrygon kuhlii* species complex [38, 72] where several species are sympatric yet cannot be diagnosed morphologically, which complicates management. Policymakers may find it difficult to implement conservation and management actions, especially if the species are sympatric, inhabit similar ecotypes, or occur in one fishing region. Without the ability to differentiate among the members of a complex, studies on their biology, ecology, or population stock for management purposes are rendered problematic due to the likelihood of misidentification and possibly overlapping information [37]. This is particularly challenging in countries or regions where access to genetic analysis is still limited and costly [73–75].

## Conclusions

Genetics is a powerful tool for detecting and differentiating cryptic species complexes; however, genetic differentiation that lacks supporting biological and ecological differentiators, may not be usefully applied to determine on which side of the population-species boundary a taxon falls. Genetic analyses from two populations of a relatively sedentary species that are separated by considerable geographical distance may show large fixed differences, supporting them being identified as separate species. In comparison, subsequent and sufficient sampling across the full range of the species complex would reveal any "intermediate" population, which would prevent bias in determining the efficacy of population versus species delimitation recommendations [76]. Therefore, the use of genetics for delimiting species should include comprehensive sampling locations to identify populations that may bridge differences between two or more distinct populations before they are interpreted as separate species.

It has been proposed that guidelines be developed for the application of genomic data to justify taxonomic boundaries before delimiting species from cryptic complexes [12]. Determining a new species based on genetic differences should be approached with caution and supported by strong justifications. We suggest some essential steps that should be taken before designating a distinct population or OTU derived from a genetic study to be a putative species. Firstly, it is important to ensure that sampling locations are representative across the range with consideration to their basic biological information (e.g., migratory or sedentary, pelagic or demersal), as speciation is likely occurring more often in species with low dispersal ability [77]. Secondly, there should be diagnostic morphological character(s) that can be used to distinguish the new species from the former species and other OTUs. Third, there are supporting morphological, habitat or behavioural characteristics that can be used to confidently extirpate the new species from a species complex. Lastly, clear information on geographic range should be included within the description of the new species to avoid ambiguity for users, be they biologists, policymakers or managers, to allow them to conduct further studies and related conservation and fishery assessments. In relation to the *Chiloscyllium punctatum* species complex, we suggest that a taxonomic revision is warranted to enhance conservation and management strategies for the species that presently reside within it.

## Methods

### Sampling and DNA extraction

Tissue samples from *Chiloscyllium punctatum* were obtained from 17 localities within the Indo-Australian region (Table 2). Samples from Thailand, Malaysia, and Indonesia (Sumatra, Java, Kalimantan, South Sulawesi, and Lombok) were sourced from local fish markets. Samples were from Moreton Bay, eastern Australia, samples from Papua New Guinea and Western Australia were provided by the University of Queensland and the national fish collection (CSIRO, Hobart). The size of the largest specimen from each location was noted, either through direct measurement of total length (*TL*) or derived from other sources of information (Table 2), as previous studies indicate that *C. punctatum* maximum body size may vary among locations [22, 27]. Length at maturity data was sourced from published materials for this region [24, 29–31].

All tissue samples were extracted using the DNA salting out procedure [79]. Total DNA concentration of each sample was measured using a Qubit 2.0 Fluorometer (Invitrogen, Carlsbad, CA, USA), with DNA extractions diluted to 50–150 ng.µl^−1^ for downstream processing.

### mtDNA phylogenetic analyses

A total of 34 tissue samples collected from 12 locations during 2017 was analysed with the mtDNA gene marker NADH dehydrogenase subunit 2 (NADH2). Samples were amplified using MyTaq DNA polymerase (Bioline Reagents, London) and primers from Naylor et al. [18] with a modified forward primer: ILEM Chilo2 (5'- AAG GAT CAC TTT GAT AGA GT- 3'), and modified reverse primer: ASNM Chilo (5′- AAC ACT TAG CTG TTA ACT AA-3′). Thermocycler conditions were set as follows: 3 min at 95 ℃ for initial denaturation, 32 cycles of amplification (30 s at 95℃, 20 s at 52℃, and 1 min at 72℃), and a final extension for 10 min at 72℃. Each 20 µl reaction mixture contained 10 µl MyTaq, 2 µl of each 10 pmol.µl^−1^ primer, 2 µl of 100–150 ng.µl^−1^ template DNA, and 4 µl ddH_2_O. Amplified products were analysed by electrophoresis (1% agarose gel with GelRed® stain) and checked using Syngene gel documentation system G:BOX F3 with GeneSys image acquisition software (Synoptics Group, Cambridge, UK). The PCR products were purified using ExoSAP-IT from USB (Cleveland, Ohio). Products were sequenced with ILEM Chilo2 and ND2 Batoid (5′-CAC TTY TGA TTA CCA GAA GT-3′) forward primers to extend the total read per individual to > 800 bp. Sequencing reactions were performed at the Australian Equine Genetics Research Centre (The University of Queensland) using Applied Biosystems BigDye Terminator v3.1 chemicals and standard procedures in 20 µl volumes and analysed with an Applied Biosystems 3730 DNA analyser.

Each chromatogram was visually checked and all sample sequences were aligned in Geneious Pro® v7.1.9 (Biomatters Ltd, Auckland, New Zealand), together with two reference sequences of *C. punctatum* from Genbank: GN2590 from Queensland (accession number JQ518745.1) and GN4446 from West Kalimantan (JQ519064.1). Modeltest analysis was used to estimate the best fit model of the nucleotide substitution for the mtDNA data set based on the lowest value of the Akaike Information Criterion (AIC) corrected for small sample size [80]. A maximum-likelihood phylogenetic tree was constructed using the Tamura-Nei 1993 model with a discrete Gamma distribution (TrN + G) as the best model (+ G parameter = 0.3851). The analyses were run using PAUP* 4.0a163 [81]. The tree topology was evaluated using the maximum likelihood heuristic search with ten random stepwise addition sequence replicates. Statistical support for branch nodes was evaluated by bootstrapping across 1000 replicates [82]. The phylogenetic tree was constructed using Dendroscope 3 [83]. Pairwise distances between sequences were calculated based on the TrN + G model with 1000 bootstrap replicates using Mega version X [84]. Haplotype networks were reconstructed to visualise the relationship among sequence data based on the median-joining network [85] using the PopArt program [86].

### Next-generation sequencing

A total of 148 extracted DNA samples (50–150 ng.µl^−1^) were genotyped with the DArT-seq approach by Diversity Arrays Technologies (DArT Pty Ltd, Canberra, Australia) using standard protocols as described by Kilian et al. [87]. A combination of methylation-sensitive restriction enzymes (P*st*I and S*ph*I) was used to digest the total DNA and detect SNP variation. Around two million sequences per sample generated from DArT pipelines were fragmented to 69 bp and then combined into clusters by the DArT clustering algorithm. The low-quality base and identical sequences were corrected and analysed by the DArT software (DArTsoft14) to produce candidate SNP markers. SNP markers were identified within each cluster by calculating parameters such as average and variance of sequencing depth, the average counts for each SNP allele and the call rate for each sequence across all samples [87, 88].

### SNPs filtering and genotyping analyses

The SNP data were filtered using the R package 'dartR' [89]. Loci with 100% repeatability (repAvg = 1.0) and without missing values (call rate = 1.0) were retained for subsequent analysis. Monomorphic loci and secondary SNPs within loci were removed, the latter to reduce the possibility of linked fragments. SNP loci with ≤ 1% minor allele frequency were removed.

Principal coordinate analysis (PCoA) was used to visualise the data with the R package 'dartR' [89, 90]. Pairwise *F*_*ST*_ values between populations along with confidence intervals and p-value were calculated based on the method by Weir and Cockerham [91] using the function *gl.fst.pop* in the 'dartR' package.

Fixed-difference analysis was applied to the filtered SNP data to identify private alleles at a locus that were not shared among sample locations as a robust indication of lack of gene flow [89]. Locations represented by fewer than five individuals were not included in the analysis as they may yield false-positive results [92]. Locations that had non-significant fixed differences were further amalgamated (p-value > 0.05). The final amalgamated groups from the significant fixed differences were designated as putative operational taxonomic units (OTUs). Analyses were conducted using the functions *gl.collapse.recursive* and *gl.collapse.pval* in ‘dartR’ [89, 92].

A phylogeny of the amalgamated fixed-differences matrix was constructed using a Fitch-Margoliash distance analysis based on the Euclidean distance. The program was run in Phylip version 3.698 (http://evolution.genetics.washington.edu/phylip.html). The filtered SNP genotype data were converted into a nexus file and concatenated into a single sequence tag per individual. Maximum-likelihood based phylogeny was estimated with RAXML v.8.2.10 [93] using the GTRGAMMA model with 1000 bootstrap replicates. The analysis was conducted on the Tinaroo High-Performance Computer system of the University of Queensland by implementing 20 CPUs for 18 h. The consensus phylogenetic tree was constructed using Dendroscope v.3.6.3 [83].

A multispecies coalescent analysis was performed to examine the species delimitation using SNAPP [90, 94] available in BEAST 2 [95]. Specifically, we tested five different species delimitation hypotheses. The first three hypotheses tested for two putative species based on different geographic groupings: (1) an Indo Malay-Indian Ocean and Australian region species; (2) Indo-Malay and eastern Indian Ocean *vs* Australian region; and (3) Indo-Malay and west coast of Sumatra *vs* Lombok and Australian region. The fourth hypothesis tested for three putative species as informed by habitat preferences (Indo-Malay *vs* eastern Indian Ocean *vs* Australian region). Lastly, the fifth hypothesis tested for four putative species, based on OTUs identified by the fixed difference analysis. As SNAPP is computationally demanding, we ran the analysis on one randomly selected individual per location for OTU_1_ and two individuals per location for the other OTUs. We repeated the analysis three times with different individuals to ensure reproducibility. The SNAPP datasets were prepared in BEAUTi with each location assigned as separate taxon and run with Markov chain Monte Carlo (MCMC) run for 2 million iterations and 500,000 burn-in. We set a gamma prior distribution with alpha = 3 and beta = 100 for a prior mean of 0.03 theta to reflect 0.3% sequence divergence as the mean divergence among OTUs. The convergence of the MCMC was inspected in Tracer v.1.7.1. and the maximum clade credibility calculated using TreeAnnotator v.2.6.2. The analysed tree set was visualised using DensiTree v. 2.2.7. [96]. The Bayes factor delimitation method (BFD*) was implemented as a plug-in to BEAST 2 to compare marginal likelihood estimates (MLE) for alternative species delimitation models [97]. We conducted a path sampling analysis of 50 steps (when the marginal likelihood estimate remained constant) with 500,000 MCMC iterations and burn-in of 50,000 iterations. The Bayes Factor (BF) was calculated by subtracting MLE values of two assigned models multiplying by two [97].

## Supplementary Information

**Additional file 1. **Distance matrix of pairwise genetic divergence of *C. punctatum* based on their geographic regions with *C. plagiosum* as an outgroup.

**Additional file 2. **Filtering steps of single nucleotide polymorphism dataset of 148 individuals *Chiloscyllium punctatum* from 16 locations using R package ‘dartR’.

**Additional file 3. **Average pairwise F_ST_ values of *C. punctatum* between sampling locations based on the SNPs dataset (bottom diagonal) with P-values at the top diagonal.

**Additional file 4. **The fixed allele differences matrix (bottom diagonal) and percentage fixed differences matrix (top diagonal) for *C. punctatum* from each sampling location.

**Additional file 5**. Matrix showing the counts of absolute-fixed allele differences (lower diagonal) and percentage fixed-differences (upper diagonal) of four OTUs derived from the amalgamation of some populations of *C. punctatum* from Indo-Australian region.

**Additional file 6. **Additional figures for alternative species trees generated from SNAPP analysis using different individuals and visualised by DensiTree.

## Data Availability

The datasets generated and/or analysed during the current study are available in the Dryad repository, https://datadryad.org/stash/share/BhbiYcNPKpY08z0qcUA-Me6wECzD19HqNLuK8fPXwpg.

## Abbreviations

AIC: Akaike information criterion
BEAST: Bayesian evolutionary analysis sampling trees
BF: Bayes factors
BFD: Bayes factor delimitation
BIN: Bintan, Riau, Indonesia
CSIRO: Commonwealth Scientific and Industrial Research Organisation
DArT: Diversity Arrays Technologies
DNA: Deoxyribonucleic acid
DoF: Department of Fisheries
EJV: East Java, Indonesia
EKL: East Kalimantan
LIPI: Lembaga Ilmu Pengetahuan Indonesia
LMB: Lombok, Indonesia
MCMC: Markov chain Monte Carlo
MLE: Marginal likelihood estimates
MUN: Muncar, Indonesia
mtDNA: Mitochondrial deoxyribonucleic acid
NADH2: Nicotinamide adenine dinucleotide hydrogenase subunit 2
OTU: Operational taxonomic unit
PAH: Pahang, Malaysia
PCoA: Principal coordinate analysis
PCR: Polymerase chain reaction
PER: Perak, Malaysia
PHU: Phuket, Thailand
PNG: Papua New Guinea
RAXML: Randomized axelerated maximum likelihood
SAB: Sabah, Malaysia
SEAFDEC: Southeast Asian Fisheries Development Center
SEQ: Southeast Queensland, Australia
SNAPP: SNP and AFLP Package for Phylogenetic analysis
SNPs: Single nucleotide polymorphisms
SUL: South Sulawesi, Indonesia
TL: Total length
TrN + G: Tamura-Nei 1993 model with a discrete Gamma distribution
WAU: Western Australia
WSA: Aceh, Indonesia
WSS: Sibolga, Indonesia
