# Supplementary material for: Delimiting cryptic species within the brown-banded bamboo shark, Chiloscyllium punctatum in the Indo-Australian region with mitochondrial DNA and genome-wide SNP approaches
Source: BMC Ecol Evol. 2021 Jun 16;21:121. doi: 10.1186/s12862-021-01852-3 (PMC8207608; doi:10.1186/s12862-021-01852-3)
Supplement: Supplementary file 1 — Additional file 1. Distance matrix of pairwise genetic divergence of C. punctatum based on their geographic regions with C. plagiosum as an outgroup. [file 12862_2021_1852_MOESM1_ESM.doc]

**Additional file 1**

TABLE S1. Distance matrix of pairwise genetic divergence of *C. punctatum* based on their geographic regions with *C. plagiosum* as an outgroup. The bottom diagonal is genetic distance based on the NADH2 gene, and the top diagonal is standard error estimates. The analysis was conducted using the maximum composite likelihood model (TrN+G) with a 1000 replicates bootstrap procedure.

|  | PHU | PAH | BIN | WKL | WJV | SUL | WSA | WSS | MUN | LMB | PNG | WAU | SEQ | *C. plagiosum* |
| --- | --- | --- | --- | --- | --- | --- | --- | --- | --- | --- | --- | --- | --- | --- |
| PHU |  | 0.002 | 0.003 | 0.002 | 0.005 | 0.003 | 0.005 | 0.005 | 0.005 | 0.005 | 0.007 | 0.006 | 0.007 | 0.026 |
| PAH | 0.007 |  | 0.001 | 0.000 | 0.005 | 0.003 | 0.005 | 0.006 | 0.005 | 0.005 | 0.006 | 0.006 | 0.006 | 0.028 |
| BIN | 0.007 | 0.001 |  | 0.001 | 0.005 | 0.004 | 0.005 | 0.006 | 0.005 | 0.006 | 0.007 | 0.007 | 0.007 | 0.030 |
| WKL | 0.006 | 0.000 | 0.001 |  | 0.004 | 0.003 | 0.005 | 0.005 | 0.005 | 0.005 | 0.006 | 0.006 | 0.006 | 0.027 |
| WJV | 0.020 | 0.015 | 0.017 | 0.014 |  | 0.006 | 0.007 | 0.008 | 0.007 | 0.007 | 0.009 | 0.008 | 0.009 | 0.030 |
| SUL | 0.012 | 0.008 | 0.010 | 0.008 | 0.023 |  | 0.005 | 0.005 | 0.005 | 0.005 | 0.006 | 0.006 | 0.006 | 0.027 |
| WSA | 0.027 | 0.025 | 0.022 | 0.025 | 0.037 | 0.023 |  | 0.001 | 0.003 | 0.003 | 0.005 | 0.004 | 0.005 | 0.028 |
| WSS | 0.025 | 0.023 | 0.021 | 0.023 | 0.037 | 0.023 | 0.003 |  | 0.003 | 0.003 | 0.005 | 0.004 | 0.005 | 0.028 |
| MUN | 0.022 | 0.021 | 0.019 | 0.021 | 0.036 | 0.023 | 0.013 | 0.010 |  | 0.003 | 0.005 | 0.004 | 0.005 | 0.028 |
| LMB | 0.024 | 0.022 | 0.022 | 0.021 | 0.037 | 0.021 | 0.010 | 0.007 | 0.009 |  | 0.004 | 0.003 | 0.004 | 0.029 |
| PNG | 0.035 | 0.032 | 0.031 | 0.032 | 0.046 | 0.030 | 0.025 | 0.023 | 0.021 | 0.018 |  | 0.003 | 0.001 | 0.029 |
| WAU | 0.032 | 0.030 | 0.029 | 0.029 | 0.043 | 0.026 | 0.017 | 0.015 | 0.016 | 0.010 | 0.009 |  | 0.003 | 0.028 |
| SEQ | 0.034 | 0.032 | 0.030 | 0.031 | 0.046 | 0.029 | 0.024 | 0.022 | 0.020 | 0.017 | 0.001 | 0.008 |  | 0.028 |
| *C. plagiosum* | 0.193 | 0.200 | 0.193 | 0.199 | 0.207 | 0.197 | 0.205 | 0.199 | 0.203 | 0.207 | 0.207 | 0.201 | 0.205 |  |
