# Supplementary material for: Delimiting cryptic species within the brown-banded bamboo shark, Chiloscyllium punctatum in the Indo-Australian region with mitochondrial DNA and genome-wide SNP approaches
Source: BMC Ecol Evol. 2021 Jun 16;21:121. doi: 10.1186/s12862-021-01852-3 (PMC8207608; doi:10.1186/s12862-021-01852-3)
Supplement: Supplementary file 2 — Additional file 2. Filtering steps of single nucleotide polymorphism dataset of 148 individuals Chiloscyllium punctatum from 16 locations using R package ‘dartR’. [file 12862_2021_1852_MOESM2_ESM.doc]

**Additional file 2**

Table S2. Filtering steps of single nucleotide polymorphism dataset of 148 individuals *Chiloscyllium punctatum* from 16 locations using R package ‘dartR’.

|  | Loci retained | Loci filtered |
| --- | --- | --- |
| Initial SNP dataset | 82 994 | 0 |
| Repeatability (repAvg = 1.0) | 68 208 | 14 786 |
| Callrate (threshold = 1.0) | 8239 | 59 969 |
| Sequence tag | 7957 | 282 |
| Monomorphic loci | 7466 | 491 |
| MAF < 0.01 | 6099 | 1367 |
