# Supplementary material for: Delimiting cryptic species within the brown-banded bamboo shark, Chiloscyllium punctatum in the Indo-Australian region with mitochondrial DNA and genome-wide SNP approaches
Source: BMC Ecol Evol. 2021 Jun 16;21:121. doi: 10.1186/s12862-021-01852-3 (PMC8207608; doi:10.1186/s12862-021-01852-3)
Supplement: Supplementary file 3 — Additional file 3. Average pairwise FST values of C. punctatum between sampling locations based on the SNPs dataset (bottom diagonal) with P-values at the top diagonal. [file 12862_2021_1852_MOESM3_ESM.doc]

**Additional file 3**

Table S3. Average pairwise *F_ST_* values of *C. punctatum* between sampling locations based on the SNPs dataset (bottom diagonal) with *P*-values at the top diagonal.

|  | **WSA** | **WSS** | **PHU** | **PER** | **BIN** | **PAH** | **WKL** | **WJV** | **EJV** | **EKL** | **SAB** | **SUL** | **LMB** | **PNG** | **WAU** |
| --- | --- | --- | --- | --- | --- | --- | --- | --- | --- | --- | --- | --- | --- | --- | --- |
| **WSA** |  | 0.000 | 0.000 | 0.000 | 0.000 | 0.000 | 0.000 | 0.000 | 0.000 | 0.000 | 0.000 | 0.000 | 0.000 | 0.000 | 0.000 |
| **WSS** | 0.268 |  | 0.000 | 0.000 | 0.000 | 0.000 | 0.000 | 0.000 | 0.000 | 0.000 | 0.000 | 0.000 | 0.000 | 0.000 | 0.000 |
| **PHU** | 0.840 | 0.806 |  | 0.000 | 0.000 | 0.000 | 0.000 | 0.000 | 0.000 | 0.000 | 0.000 | 0.000 | 0.000 | 0.000 | 0.000 |
| **PER** | 0.817 | 0.777 | 0.268 |  | 0.000 | 0.000 | 0.000 | 0.000 | 0.000 | 0.000 | 0.000 | 0.000 | 0.000 | 0.000 | 0.000 |
| **BIN** | 0.868 | 0.852 | 0.508 | 0.105 |  | 0.097 | 0.023 | 0.000 | 0.000 | 0.000 | 0.000 | 0.000 | 0.000 | 0.000 | 0.000 |
| **PAH** | 0.870 | 0.854 | 0.512 | 0.105 | 0.003 |  | 0.362 | 0.000 | 0.000 | 0.000 | 0.000 | 0.000 | 0.000 | 0.000 | 0.000 |
| **WKL** | 0.863 | 0.844 | 0.499 | 0.099 | 0.006 | 0.001 |  | 0.003 | 0.036 | 0.000 | 0.000 | 0.000 | 0.000 | 0.000 | 0.000 |
| **WJV** | 0.860 | 0.841 | 0.499 | 0.106 | 0.013 | 0.011 | 0.009 |  | 0.000 | 0.000 | 0.000 | 0.000 | 0.000 | 0.000 | 0.000 |
| **EJV** | 0.846 | 0.819 | 0.471 | 0.094 | 0.014 | 0.011 | 0.005 | 0.014 |  |  | 0.000 | 0.000 | 0.000 | 0.000 | 0.000 |
| **EKL** | 0.815 | 0.772 | 0.425 | 0.092 | 0.043 | 0.041 | 0.033 | 0.039 | 0.017 |  |  | 0.000 | 0.000 | 0.000 | 0.000 |
| **SAB** | 0.763 | 0.697 | 0.398 | 0.243 | 0.289 | 0.290 | 0.277 | 0.279 | 0.247 | 0.199 |  |  | 0.000 | 0.000 | 0.000 |
| **SUL** | 0.814 | 0.775 | 0.640 | 0.610 | 0.672 | 0.675 | 0.665 | 0.663 | 0.636 | 0.577 | 0.437 |  |  | 0.000 | 0.000 |
| **LMB** | 0.858 | 0.847 | 0.847 | 0.823 | 0.862 | 0.864 | 0.858 | 0.856 | 0.845 | 0.820 | 0.781 | 0.819 |  |  | 0.000 |
| **PNG** | 0.748 | 0.618 | 0.712 | 0.706 | 0.749 | 0.750 | 0.743 | 0.748 | 0.726 | 0.695 | 0.637 | 0.695 | 0.786 |  |  |
| **WAU** | 0.876 | 0.830 | 0.801 | 0.775 | 0.852 | 0.855 | 0.844 | 0.842 | 0.817 | 0.770 | 0.683 | 0.765 | 0.874 | 0.082 |  |
| **SEQ** | 0.863 | 0.837 | 0.839 | 0.829 | 0.857 | 0.858 | 0.854 | 0.854 | 0.844 | 0.825 | 0.792 | 0.821 | 0.869 | 0.615 | 0.750 |
