# Supplementary material for: Delimiting cryptic species within the brown-banded bamboo shark, Chiloscyllium punctatum in the Indo-Australian region with mitochondrial DNA and genome-wide SNP approaches
Source: BMC Ecol Evol. 2021 Jun 16;21:121. doi: 10.1186/s12862-021-01852-3 (PMC8207608; doi:10.1186/s12862-021-01852-3)
Supplement: Supplementary file 4 — Additional file 4. The fixed allele differences matrix (bottom diagonal) and percentage fixed differences matrix (top diagonal) for C. punctatum from each sampling location. [file 12862_2021_1852_MOESM4_ESM.doc]

**Additional file 4**

Table S4**.** The fixed allele differences matrix (bottom diagonal) and percentage fixed differences matrix (top diagonal) for *C. punctatum* from each sampling location. Due to the low sample size, WSS and WAU (marked in red) were amalgamated to the closest geographical location (WSA and PNG, respectively).

|  | BIN | PAH | PER | WKL | WJV | EJV | EKL | SAB | PHU | SUL | LMB | WSA | WSS | PNG | WAU | SEQ |
| --- | --- | --- | --- | --- | --- | --- | --- | --- | --- | --- | --- | --- | --- | --- | --- | --- |
| BIN |  | 0 | 0 | 0 | 0 | 0 | 0 | 0 | 0 | 3 | 13 | 15 | 15 | 17 | 9 | 21 |
| PAH | 0 |  | 0 | 0 | 0 | 0 | 0 | 0 | 0 | 3 | 13 | 15 | 15 | 10 | 17 | 21 |
| PER | 0 | 0 |  | 0 | 0 | 0 | 0 | 0 | 0 | 3 | 13 | 14 | 14 | 9 | 16 | 20 |
| WKL | 0 | 0 | 0 |  | 0 | 0 | 0 | 0 | 0 | 3 | 13 | 14 | 14 | 9 | 16 | 20 |
| WJV | 0 | 0 | 0 | 0 |  | 0 | 0 | 0 | 0 | 3 | 12 | 14 | 14 | 9 | 16 | 20 |
| EJV | 0 | 0 | 0 | 0 | 0 |  | 0 | 0 | 0 | 2 | 11 | 13 | 13 | 8 | 15 | 19 |
| EKL | 0 | 0 | 0 | 0 | 0 | 0 |  | 0 | 0 | 1 | 10 | 11 | 12 | 6 | 13 | 17 |
| SAB | 0 | 0 | 0 | 0 | 0 | 0 | 0 |  | 0 | 0 | 10 | 12 | 12 | 7 | 14 | 17 |
| PHU | 8 | 8 | 0 | 6 | 8 | 7 | 7 | 9 |  | 3 | 14 | 15 | 15 | 9 | 17 | 21 |
| SUL | 184 | 201 | 153 | 171 | 162 | 97 | 38 | 15 | 206 |  | 12 | 14 | 14 | 7 | 15 | 18 |
| LMB | 796 | 821 | 766 | 773 | 753 | 688 | 584 | 639 | 846 | 742 |  | 13 | 13 | 10 | 17 | 21 |
| WSA | 886 | 899 | 830 | 851 | 843 | 780 | 682 | 706 | 899 | 833 | 775 |  | 0 | 10 | 19 | 22 |
| WSS | 917 | 931 | 861 | 879 | 871 | 804 | 710 | 736 | 929 | 856 | 773 | 9 |  | 11 | 19 | 23 |
| PNG | 571 | 581 | 522 | 546 | 537 | 480 | 389 | 397 | 568 | 457 | 601 | 633 | 660 |  | 1 | 2 |
| WAU | 1024 | 1043 | 962 | 994 | 987 | 913 | 805 | 826 | 1036 | 902 | 1067 | 1148 | 1187 | 34 |  | 14 |
| SEQ | 1254 | 1272 | 1194 | 1224 | 1217 | 1143 | 1032 | 1043 | 1264 | 1127 | 1287 | 1349 | 1429 | 149 | 841 |  |
