# Supplementary material for: Delimiting cryptic species within the brown-banded bamboo shark, Chiloscyllium punctatum in the Indo-Australian region with mitochondrial DNA and genome-wide SNP approaches
Source: BMC Ecol Evol. 2021 Jun 16;21:121. doi: 10.1186/s12862-021-01852-3 (PMC8207608; doi:10.1186/s12862-021-01852-3)
Supplement: Supplementary file 5 — Additional file 5. Matrix showing the counts of absolute-fixed allele differences (lower diagonal) and percentage fixed-differences (upper diagonal) of four OTUs derived from the amalgamation of some populations of C. punctatum from Indo-Australian region. [file 12862_2021_1852_MOESM5_ESM.doc]

**Additional file 5**

Table S5. Matrix showing the counts of absolute-fixed allele differences (lower diagonal) and percentage fixed-differences (upper diagonal) of four OTUs derived from the amalgamation of some populations of *C. punctatum* from Indo-Australian region.

| Group of populations | No. indiv |  | OTU_1_ | OTU_2_ | OTU_3_ | OTU_4_ |
| --- | --- | --- | --- | --- | --- | --- |
| (PER, PAH, BIN, WKL, WJV, EJV, EKL, SAB, PHU, SUL) | 103 | OTU_1_ |  | 6 | 3 | 6 |
| LMB | 16 | OTU_2_ | 343 |  | 11 | 8 |
| (WSA, WSS) | 10 | OTU_3_ | 381 | 684 |  | 8 |
| (PNG, SEQ, WAU) | 19 | OTU_4_ | 189 | 474 | 462 |  |
