# Supplementary material for: Delimiting cryptic species within the brown-banded bamboo shark, Chiloscyllium punctatum in the Indo-Australian region with mitochondrial DNA and genome-wide SNP approaches
Source: BMC Ecol Evol. 2021 Jun 16;21:121. doi: 10.1186/s12862-021-01852-3 (PMC8207608; doi:10.1186/s12862-021-01852-3)
Supplement: Supplementary file 6 — Additional file 6. Additional figures for alternative species trees generated from SNAPP analysis using different individuals and visualised by DensiTree. [file 12862_2021_1852_MOESM6_ESM.doc]

**Additional file 6**

**Supplementary Figures**


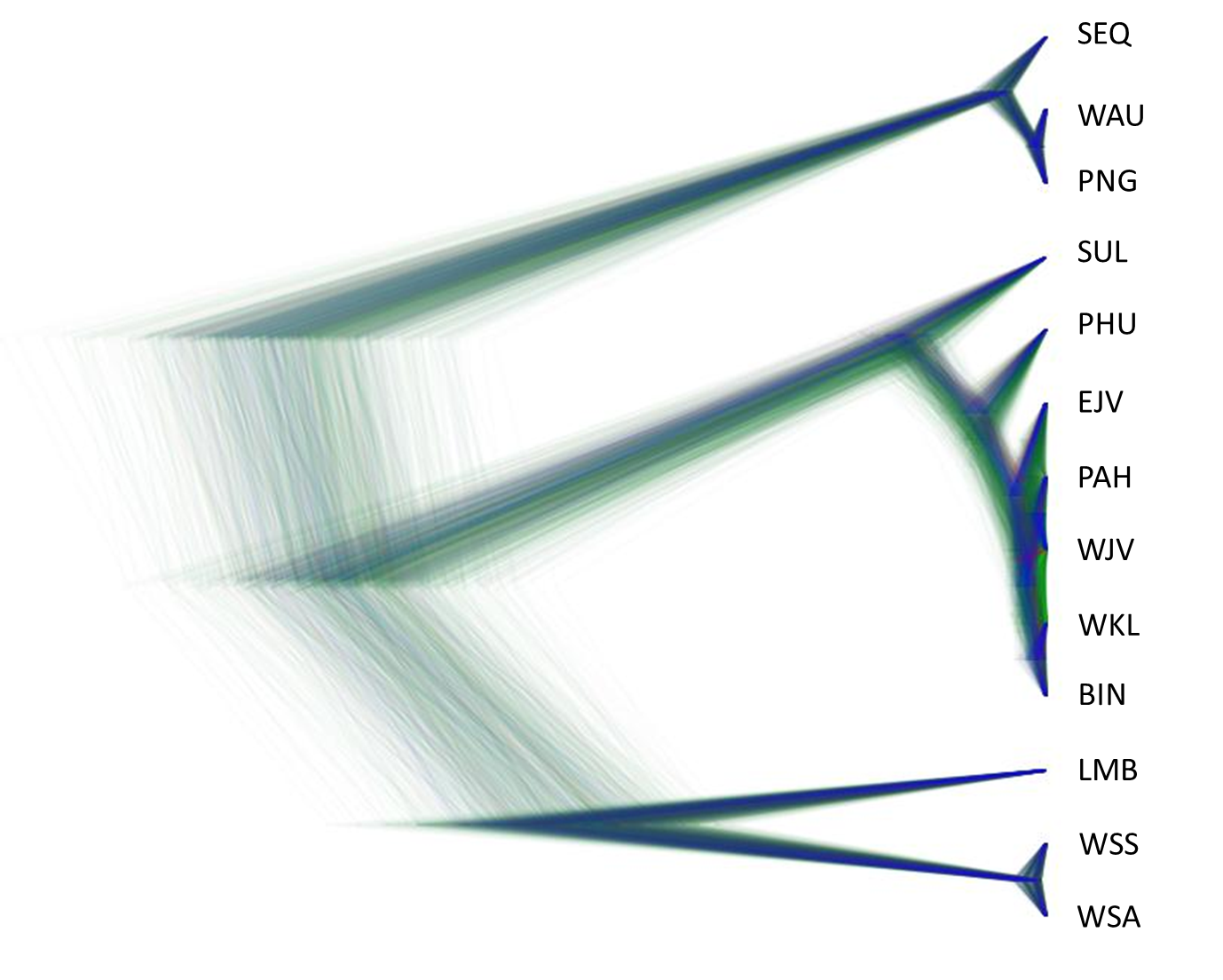


FIGURE S1**.** The species trees of 16 individuals representing 13 sampling locations from SNAPP analysis taken from every 1000 steps of the total 2,000,000 iterations visualised by DensiTree software.


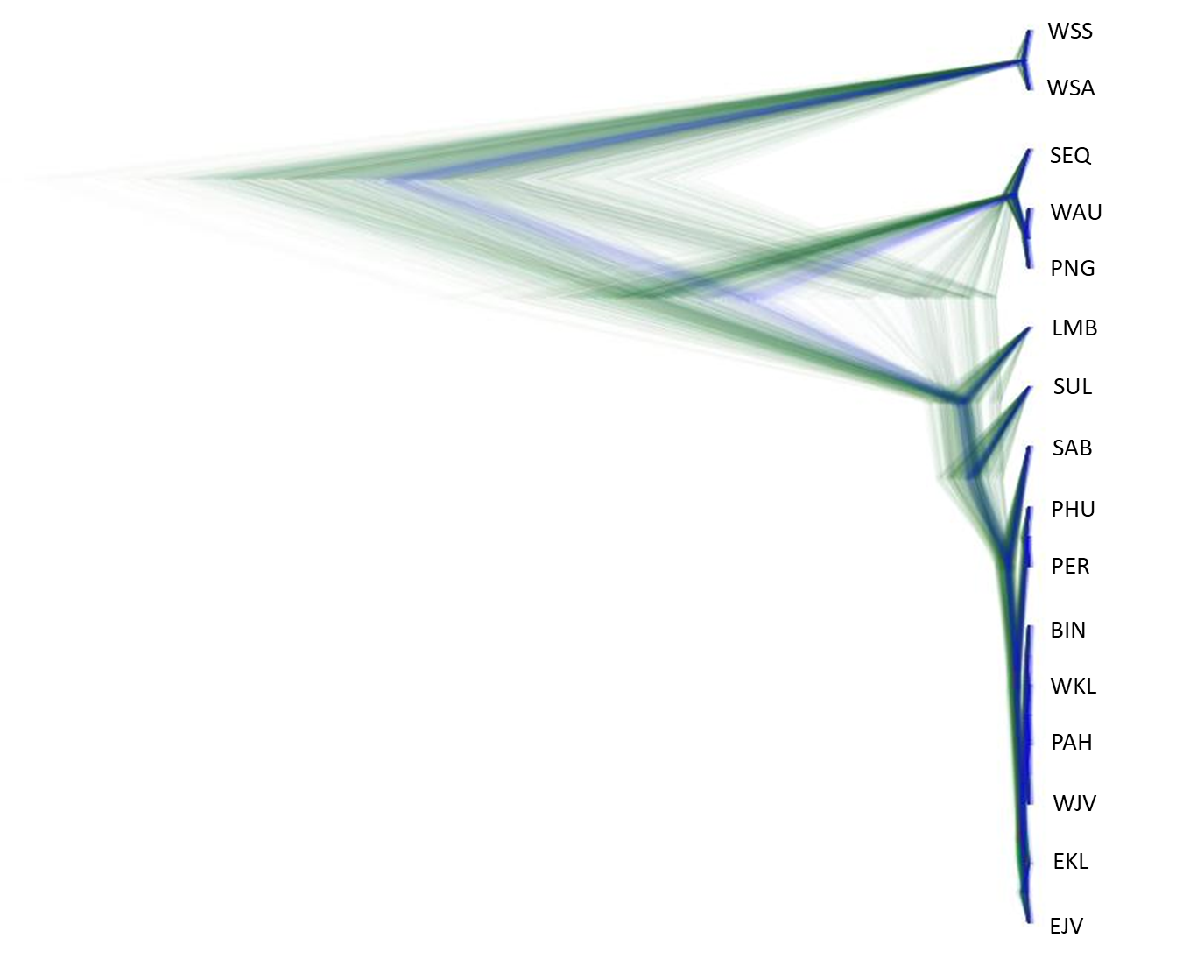


FIGURE S2**.** The species trees of 20 individuals representing 16 sampling locations from SNAPP analysis using a different dataset.
